# Application of Unprocessed Waste Tyres in Pavement Base Structures: A Study on Deformation and Stress Analysis Using Finite Element Simulation

**DOI:** 10.3390/ma18040914

**Published:** 2025-02-19

**Authors:** Baoying Shen, Hui Tian, Wenruo Fan, Lu Zhang, Hui Wang

**Affiliations:** 1 Chongqing Architectural Design Institute Co., Ltd., Chongqing 400015, China; shenbaoying111@foxmail.com (B.S.); tianhui333@foxmail.com (H.T.); alana66@foxmail.com (L.Z.); 2 School of Civil Engineering, Chongqing University, Chongqing 400045, China; 15080868861@163.com

**Keywords:** waste tyre, base layer, deformation resilience, finite element simulation

## Abstract

In this study, numerical simulations using the Abaqus finite element model were performed to evaluate the effects of incorporating waste tyres of varying sizes into the base layer as part of a coupled tyre–pavement structure. The tyre-reinforced structure demonstrated superior deformation resilience, attributed to the hyperelastic properties of tyre rubber, underscoring its potential for applications where deformation recovery is essential. For achieving a uniform settlement, the entire tyre stacking scheme is recommended, whereas the one-third tyre configuration is ideal for minimising displacement. The one-half tyre configuration provides a balanced approach, optimising resource utilisation for structures with moderate performance requirements. The inclusion of tyres increases the equivalent stress within the cement-stabilised gravel layer beneath the tyre, and this effect is less pronounced with smaller tyre sizes. Notably, the projected portion of the tyre tread enhances the bearing capacity of the base structure, improving the load distribution and overall structural performance. The middle and bottom surface layers were identified as the most critical for controlling deformation and stress distribution, while a moderate modulus is advised for the surface course to achieve a balance between deformation control and stress uniformity. The integration of high-modulus layers with tyre reinforcement offers an optimised solution for both deformation management and stress distribution. This study highlights the potential of tyre-reinforced pavements as an innovative and sustainable construction practice, particularly suited for light to moderate traffic conditions. Further research is recommended to explore the long-term environmental and economic benefits, as well as the impacts of tyre composition and ageing on performance.

## 1. Introduction

The accelerated development of the automotive industry, coupled with widespread consumer use, has led to a significant escalation in the generation of waste tyres. These tyres have become a major concern due to their significant resource and environmental impacts. The utilisation of these waste tyres in various forms within the engineering and construction sector has been a subject of considerable attention [1,2,3,4,5]. In 2020, the total number of new tyres sold in Europe amounted to 324 million units [6]. In China, the generation of waste tyres reached approximately 330 million in 2019, equivalent to more than 10 million tonnes in weight [7]. With the projected increase in car ownership to 340 million by the end of 2023 [8], it is anticipated that the amount of waste tyres that will be produced each year will increase. Projections by Cui et al. [5] indicate that China’s car ownership will continue to grow and reach saturation by 2045, with private cars and heavy trucks accounting for more than 78% of future waste tyre production. Consequently, the large-scale engineering application of used tyres is, therefore, identified as a key solution to address tyre pollution.

The utilisation of waste tyres as an auxiliary raw material in various civil engineering applications is attributable to their light weight, compressibility, vibration, and noise-reduction properties [9,10,11]. A substantial amount of technical literature, encompassing laboratory and field tests, implementation projects, and specific standards, has been published [10,11,12,13,14,15,16]. Nevertheless, substantial deficiencies in the recycling of tyres endure, with a significant proportion of tyres still necessitating disposal in deep landfills. Research has thus far focused on two main application scenarios: rubber-modified asphalt [9] and tyre-derived aggregates (TDAs) [10,11,12]. The potential exists for processed tyres to contaminate drinking water when used in the subgrade, but distance dilution is sufficient to address this issue [15]. The predominant market for ground tyre rubber in America is asphalt rubber, with a consumption of approximately 12 million tyres [17]. Karim et al. [18] attempted to incorporate the secondary use of the sub-base in the form of rubber granules, and they found that with the variation in the design thickness of the structural layer caused by different additive proportions of rubber, additions above 1% brought about a significant reduction in strength, requiring an increase in the thickness of the surface layer to meet the design requirements, thus increasing the construction cost. A pioneering study [19] has been conducted that analysed the use of coarser TDAs (4.75–2.36 mm) in the manufacture of rubberised asphalt mixtures as an alternative to traditional unbound or cement-treated mixtures as a base layer. The study confirmed the advantages of TDA additions, which can be as high as 1–2% by weight. However, this particle scale is more expensive to apply and can cause contamination due to the abrasion and precipitation of material from the rubber particles.

A more environmentally sustainable approach involves the direct utilisation of low-processed tyres, including scrap tyres [20], buried full tyres [21], and tyre-tied tyre bales [22]. The utilisation of whole tyres as reused construction material in the reuse process constitutes a high-value recycling option that demands fewer resources and less energy than recycling (e.g., grinding) and is consistent with the large-scale implementation of the principles of a circular economy [23]. Consequently, the prioritisation of tyre reuse over recycling after mechanical or chemical treatment can facilitate the enhancement of tyre production and consumption systems to become more environmentally friendly [24]. Research on the interaction of complete tyres (tyre bales) with the natural environment has shown that complete tyres have little or no negative impact on the natural environment [25]. These low-processed tyres were primarily applied in structural elements such as retaining walls and base foundations [4,20,21,22]. As urban development progresses, the era of large-scale urban construction has transitioned, and the availability of land for the burial of tyres, where large-scale applications have yet to be implemented, is also diminishing substantially. Consequently, highway structure construction, characterised by its extensive mileage, substantial burial space, and the necessity for vegetation isolation, has emerged as a pivotal application scenario for integrally buried tyres [16]. The utilisation of tyres in upper structures (e.g., base), as opposed to the subgrade, may constitute a comparatively sustainable scenario for tyre recycling. The utilisation of lightly treated intact tyres for pavement structural layers has the potential to offer a sustainable, low-pollution engineering application scenario for tyre recycling.

The design of new pavement structures was commonly analysed and validated using the finite element model (FEM) simulation method [26,27,28,29]. An analysis by Cortes et al. [26] of inverted structures revealed that the linear elastic analysis method could be utilised to simulate the limiting conditions. In a structural layer simulation by Jiang et al. [28], the linear elastic response of the structural layers, excluding the unconfined aggregates, was employed. The validity of the simulations for damage, deformation, and stress distribution can be verified through actual road tests. There are significant discrepancies between the material parameters employed in laboratory tests and an actual road surface [29], which complicates the achievement of a structural analysis of complete tyre utilisation directly using scaled-down simulations.

The present study posits the undertaking of a numerical simulation study of tyre application to the base layer. This will be based on the fundamental assumptions of structural material parameters obtained from extant whole-tyre and tyre bale reuse studies. The aim is to determine the cutting and set-up programme of tyres.

## 2. Numerical Modelling and Material Parameter Setting

### 2.1. Numerical Model of the Pavement Structure

The linear elasticity model has been proven to be capable of being used in the study of asphalt pavement structures under loads, including deformation, stress, and damage [28]. The isotropic elasticity model was selected for utilisation as the intrinsic model, representing one of the most extensively employed models grounded in the generalised Hooke’s law. The linear elastic model’s eigen-structure equation is expressed in (1):

(1) $$\sigma =D^{el}\varepsilon^{el}$$

where σ represents the component vector of stresses, $D^{el}$ represents the elasticity matrix, and $\varepsilon^{el}$ represents the component vector of strain.

Poisson’s ratio (*ν*) and Young’s modulus (*E*) are important parameters of isotropic linear elasticity models, and the elastic parameters can be defined as a function of field variables (e.g., temperature). The shear modulus *G* can be expressed in terms of *E* and *ν*, as in (2).

(2) $$G=\frac{E}{2\;(1+\nu )}$$

For the three-dimensional case, Hooke’s law can be expressed as (3).

(3) ξ11ξ22ξ33γ12γ13γ23=1E−νE−νE000−νE1E−νE000−νE−νE1E0000001G0000001G0000001Gσ11σ22σ33σ12σ13σ23

The thickness of the road surface layer, in conjunction with the material properties of each structural layer, exerts a degree of influence on the mechanical response of the road structure to varying extents. In the simulation of this study, the material properties of asphalt mixture were used for the pavement structure, and the pavement structure was simplified from top to bottom into two main functional layers, namely the surface layer and the base layer. The establishment of the three-dimensional pavement structure model was achieved by implementing the build module, which involves the stretching of the two-dimensional plane sketch of the road structure layer and forming the assumed depth of the structural layer.

**Figure 1:**
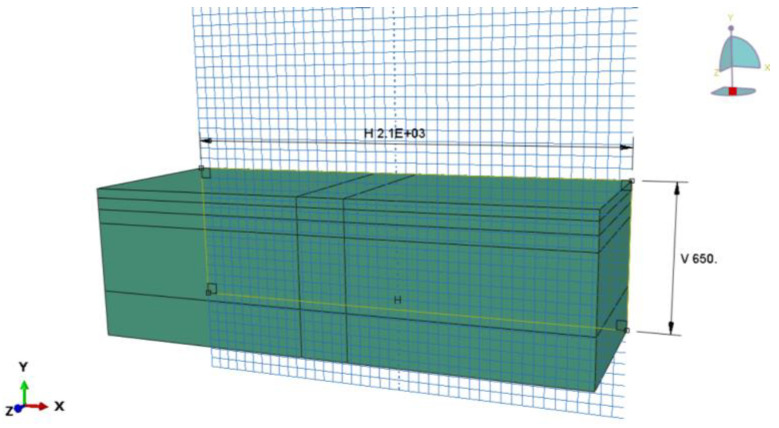
A 3D model of the pavement structure.

The initial structural dimensions of the surface layer are 2100 mm × 800 mm × 150 mm (length × width × height), including the upper surface layer, the middle layer, and the lower layer, with thicknesses of 40 mm, 50 mm, and 60 mm respectively, totalling 150 mm. The structural dimensions of the base layer are 2100 mm × 800 mm × 500 mm (length × width × height), including a cement-stabilised gravel layer and a cement-stabilised soil layer, with the thickness of each layer of 300 mm and 200 mm, respectively, which has been widely used in highway construction in China. The three-dimensional model of each structural layer of the road is shown in Figure 1, with the axes corresponding to the cross-section direction (X-axis), road vehicle travelling direction (Z-axis), and road surface depth direction (Y-axis). The main structural material parameters are shown in Table 1.

### 2.2. Numerical Model of Tyre

Notwithstanding the utilisation and the process of degradation undergone by waste tyre treads, their primary constituent is still rubber or rubber compounds, which consequently retain specific superelastic properties. In finite element simulation, the strain energy function is generally used to describe the hyperelastic constitutive relationship of rubber materials, and its general formula is (4):

(4) $$U=U(I_{1},{\;I}_{2}\;,I_{3})$$

where *U* is the strain potential energy, which represents the strain energy stored per unit volume of the material, and *I*_1_, *I*_2_, and *I*_3_ are mechanical invariants, respectively. In Abaqus/CAE 2024 software, the strain energy function was corrected using Equation (5):

(5) $$U=U_{1}\left({\bar{I}}_{1}-3,{\bar{I}}_{2}-3\right)+U_{2}(J_{el}-1)$$

where *U*_1_ and *U*_2_ denote the responses to volumetric and deviatoric deformation, respectively, and *J_el_* represents the elastic volume ratio.

A study was conducted to compare nine classes of hyperelastic material models [30]. The findings demonstrated that both the binomial polynomial and Yeoh models exhibited stability in predicting uniaxial tension over a wide range, from 0 to 1.5. For in-plane shear, all nine models accurately predicted material behaviour up to 0.5, the Marlowe model predicted the response over the strain range up to 1, and the reduced polynomial model also matched the experimental results over the strain range between 1.1 and 1.6. Consequently, the reduced polynomial model was selected as the intrinsic model for tyres, and its general formula is (6):

(6) $$U=\sum_{i=1}^{N}C_{i0}{\left({\bar{I}}_{1}-3\right)}^{i}$$

where *C_i_*_0_ is the material parameter and the Neo–Hook model when *N* = 1; the model equation is (7).

(7) $$U=C_{10}({\bar{I}}_{1}-3)$$

In the absence of the consideration of the tread pattern, tyres are regarded as regular, perfectly axisymmetric objects. Any cross-section through the tyre’s axis of symmetry is the same. This property can be employed to create a model in Abaqus Revolve, i.e., rotational modelling. The subsequent meshing and contact calculations are simplified by the simplification of the curved ‘C’ of the tyre tread to ‘]’ for ease of modelling. The tyre model was set to be 195/55/R16 85V, i.e., a radial tyre with a width of 195 mm, a tyre aspect ratio of 55 (the section height is 55% of the width), and a rim diameter of 15 inches (approximated at 380 mm).

**Figure 2:**
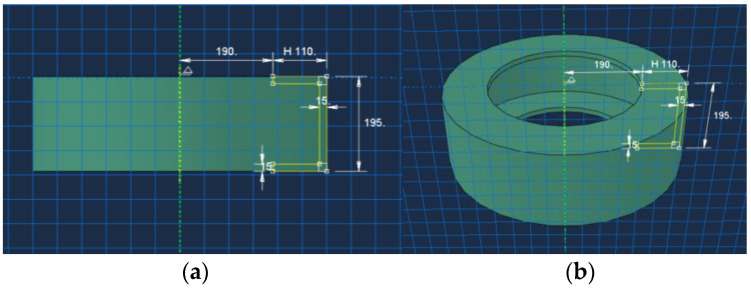
Tyre modelling. (**a**) Planar parameters; (**b**) 3D modelling.

The three-dimensional model of the tyre is shown in Figure 2. In this model, *C*_10_ was set as 0.5593 based on engineering experience.

## 3. Structural Model of Tyre–Road Coupling

### 3.1. Tyre Arrangement Method

**Figure 3:**
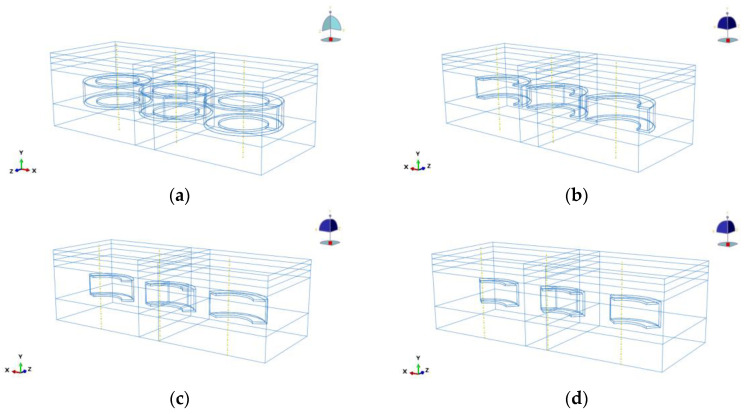
Tyre arrangement method. (**a**) Whole; (**b**) half; (**c**) one-third (third); (**d**) one-quarter (quarter).

To optimise the waste tyre stacking scheme, the tyres were categorised into four distinct types: complete tyre, 1/2 tyre, 1/3 tyre, and 1/4 tyre, as illustrated in Figure 3. The number of placements was designated as either 1 or 3, thus yielding a total of eight placement scenarios. The forces exerted by these eight models were analysed by comparing them with the force conditions of the pavement structure model without tyres, resulting in a total of nine scenarios. During the placement process, the lowermost extremity of the tyre corresponded to the lowermost extremity of the cement-stabilised gravel layer, i.e., at 450 mm below the surface. It is imperative to ensure that the centre of gravity of tyres of different scales is as close as possible to the same position. To avoid stress concentrations or stress overlaps when placing multiple tyres, tyre spacing should be set at 1.5–2 times the tyre diameter. It is imperative to reduce the spacing in consideration of the load-bearing capacity, thereby enhancing support. Moreover, for base materials characterised by low stiffness, it is essential to reduce the spacing to avert uneven settlement. In conclusion, the distance between adjacent tyre centres was set at 650 mm for this simulation study.

### 3.2. Contact Surfaces and Interactions

In the context of a waste rubber tyre-constructed base layer, it is imperative to consider the relative sliding and disengagement of the interface between the rubber tyre and the base when subjected to vertical loads. When the rubber tyre and base are in contact with each other, the force perpendicular to the contact surface of the rubber tyre and the base will act on these two objects. In the event of friction between the contact surfaces of the two objects, tangential shear force may be generated to prevent misalignment. It is, therefore, evident that the mechanical properties of the rubber tyre–road interface are highly intricate. This paper assumed the rubber tyre–road contact interface can be transferred simultaneously with tangential stresses and normal stresses. In the finite element model developed using Abaqus, the normal contact relationship between the rubber tyre and the base contact surface was governed by the ‘hard contact’ formula, while the tangential contact relationship was modelled as a ‘penalty’ friction formula. The friction coefficient of 0.3 was determined through a simulation test calculation. The calculation was simplified by defining the intersection interface between the rectangular base and the discarded rubber tyre structural foundation as no relative sliding.

### 3.3. Experimental Settings

#### 3.3.1. Loading Conditions

In this study, the loads were categorised into two cases: a static homogeneous load and moving static homogeneous load. Static homogeneous loading signifies that the force acting on the structure is uniformly distributed in a certain area and does not vary with time. This form of loading is primarily employed to observe the variation of the mechanical response of a road structure along space in the base case. Specifically, it facilitates an understanding of the distribution of stresses and strains at different locations, thereby enabling the assessment of the overall performance and stability of the road structure. Mobile static homogeneous loading signifies that the forces acting on the structure are not only uniformly distributed but also vary with time and location. This form of loading can more realistically simulate the dynamic effects of actual traffic situations. Moving static homogeneous loading has been employed to observe the mechanical response of a road structure under a moving constant load over time, thereby facilitating an analysis of the distribution of stresses and strains at different locations during vehicle travel and enhancing the comprehension of the behaviour of road structures under dynamic loading conditions. The mobile static homogeneous load is well suited for simulating the effects on road structures during vehicle travel, including phenomena such as vibration and deformation caused by vehicle passing.

**Figure 4:**
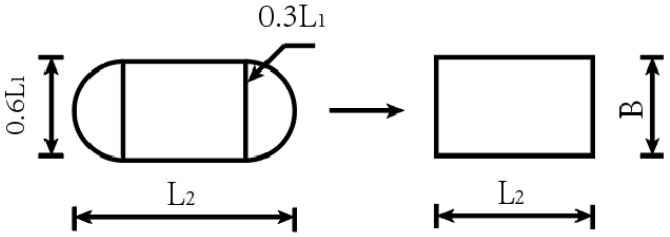
Conversion of tyre–road contact shape and equivalent area.

In the finite element static load simulation, the mechanical response of the pavement structure under static load is investigated by specifying the contact pressure of tyres with the pavement structure, the press contact surface contact, and the press contact position. The standard design load for the double wheel was set to a single axle, the wheel load P was 100 KN, and the tyre pressure strength was 0.7 Mpa. The following factors were not considered during the process of calculation and analysis: tyre type, tread pattern, and age. Concerning the contact area analysis, a combined shape consisting of semicircles was used to approximate the shape of each wheel in contact with the road surface. This is often converted into a regular rectangle utilising an equivalent area to facilitate modelling (see Figure 4). The calculations are based on Equations (8)–(10).

(8) $$A_{c}=\pi (0.3l{)}^{2}+\left(0.4l\right)\left(0.6l\right)=(0.09\pi +0.24)l^{2}$$

(9) $$L_{1}=\sqrt{\frac{A_{c}}{0.09\pi +0.24}}=\sqrt{\frac{F/p}{0.09\pi +0.24}}$$

Based on the equivalent area conversion, the ellipse area can be further simplified to a single rectangle of equal width (*L*_2_ × *B*).

(10) $$L_{2}=\frac{A_{c}}{B}=\frac{0.09\pi +0.24}{0.6}L_{1}$$

Following a thorough calculation process, the following dimensions were obtained: *L*_2_ = 228 mm and *B* = 157 mm.

To accentuate the mechanical response of the homogeneous static load, the full-length arrangement was selected. This arrangement entailed the placement of a 200 mm wide strip at the centre, with a pressure of 0.7 MPa applied along the entire Z-direction length. In the case of a moving uniform load, a moving constant load with a range of 228 mm (Z-direction) × 157 mm (X-direction) was chosen to move from the position of Z = 0 to the position of Z = 650 mm at a speed of 20 km/h.

#### 3.3.2. Boundary Condition Setting

It is imperative to recognise the profound impact of boundary conditions on the calculation outcomes, underscoring the necessity for the judicious selection of appropriate boundary conditions. In this study, the bottom surface does not extend to the depth of influence of the soft soil roadbed under the action of traffic load. It can, therefore, be set that the displacement of the bottom surface in all directions was subject to constraints. Due to the symmetry of the structure, the side surface was set to be symmetrical constraints. Similarly, the displacement in the X-direction and the rotation of the Y-axis and Z-axis were restricted in the perpendicular direction of travel. The structural part of the road was a free boundary, and the corresponding settings were as follows:

(1) The bottom surface of the road model was completely fixed (U_1_ = U_2_ = U_3_ = U_R1_ = U_R2_ = U_R3_ = 0);
(2) The left and right sides of the road model were symmetric about the plane perpendicular to the X-axis (U_1_ = U_R2_ = U_R3_ = 0);
(3) The front and rear faces of the road model were symmetric about the plane perpendicular to the Z-axis (U_3_ = U_R1_ = U_R2_ = 0).

#### 3.3.3. Model Meshing and Calculation Assumptions

It is widely acknowledged that employing a solitary cell type for meshing is generally considered ill advised, as this may yield outcomes that are less precise and more unstable. Instead, the judicious selection of a combination of cells is advocated, based on the characteristics of the particular problem, to ensure the validity and reliability of the analysis. It is important to note that hyperelastic materials characteristically exhibit significant geometrically nonlinear behaviour, which means that a solitary cell type may not accurately capture the stress and strain states during complex deformation processes. In such cases, it is necessary to employ cell types that are better equipped to manage substantial deformations and high-strain gradients. It is, therefore, the case that a single unit type may not be adequate to meet these requirements, and consequently, an appropriate combination of unit types must be selected based on the particular problem at hand.

**Figure 5:**
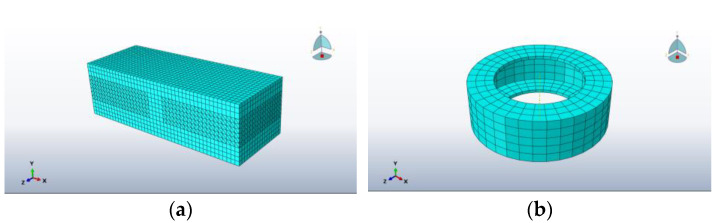
Grid division schematic diagram. (**a**) Road grid division; (**b**) tyre grid division.

In this instance, the cement-stabilised gravel layer was selected for meshing using C3D10 (three-dimensional ten-node quadratic hexahedral cell) due to the presence of the gap created by the tyre reservation position. The tyre was meshed using C3D8RH (eight-node linear reduced-integral hexahedral cell), and the remaining road structural layer were meshed using C3D8R (eight-node linear hexahedral cell) with the cell mesh. The meshing diagrams are shown in Figure 5. The total number of cells for the coupled tyre–road structure with three complete tyre faces placed was 36,875.

The calculation assumptions for the tyre–pavement structures are as follows:

(1) The constructional stress and structural self-weight were not considered, and the initial displacement generated was considered to be produced by external loads;
(2) Under the action of traffic load, the surface layer and base material of the road were completely elastic deformation;
(3) The deformation between the layers of the road structure was continuous, with neither relative sliding nor relative separation;
(4) The displacement between the base and the roadbed in all directions was zero, the displacement of the lateral horizontal direction was zero, and the displacement of the road surface was free;
(5) The elastic parameters of the road and the hyperelastic parameters of the rubber remained unchanged during each loading;
(6) Temperature changes were not considered.

## 4. Sensitivity Analysis of Pavement Structural Modulus

Changes in the moduli of the pavement’s structural layers have been demonstrated to significantly influence the structural performance of the pavement. To investigate this effect, a sensitivity analysis was conducted, focusing on two key indicators: the maximum deformation at the bottom of the surface layer (MDBSL) and the extreme difference of equivalent stress in the depth direction (EDESDD). The modulus parameters of each structural layer of the pavement were selected as the influencing factors for this analysis. The test was performed using a combination of three complete tyres to simulate realistic loading conditions. The parameter design adopted four levels, as detailed in Table 2, while the orthogonal experimental design used to systematically evaluate the factors is presented in Table 3. This approach ensures a comprehensive assessment of the impact of modulus variations on pavement performance.

## 5. Results and Discussion

### 5.1. Simulated Results Under Static Loads

#### 5.1.1. Structural Displacement Analysis for Static Loads

**Figure 6:**
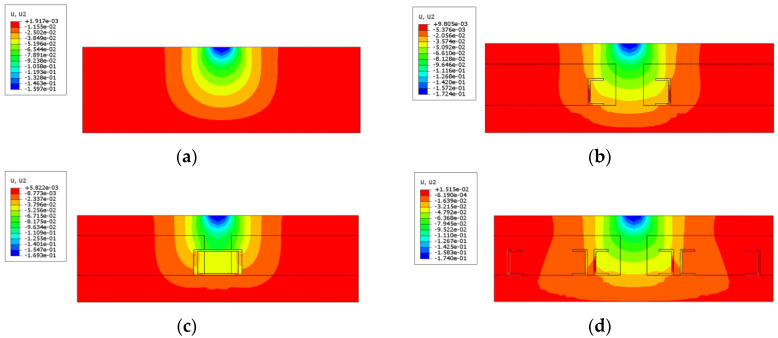
Typical pavement structure deformation maps. (**a**) Original road; (**b**) one whole tyre placed; (**c**) one 1/4 tyre placed; (**d**) three whole tyres placed.

Typical tyre–pavement structure deformation maps are shown in Figure 6.

As illustrated in Figure 6a, the presence of tyres results in a more homogeneous distribution of the structure’s displacement after loading. Furthermore, a comparison of Figure 6b,c reveals that as the size of the placed tyre decreases, the displacement distribution becomes less uniform, approaching the state of the original road surface. The displacement in Figure 6b is higher than in the other cases due to the concentration of stresses caused by the structure modelled with more mutations, resulting in sudden changes in the positive maximum of the displacements. Conversely, Figure 6c demonstrates that the internal position of the tyre is closer to the inner wall of the tyre. This is due to the tyre’s hoop effect, which distributes displacement uniformly and reduces deformation. This, in turn, enhances the roadbed’s resistance to deformation. A comparison of Figure 6b,d reveals that the number of tyres has a negligible effect on the maximum and minimum values of displacement under these loading conditions. It is only when multiple tyres are placed simultaneously that the phenomenon of expanding the range of displacement uniformity can be achieved in the cement gravel-stabilised layer.

**Figure 7:**
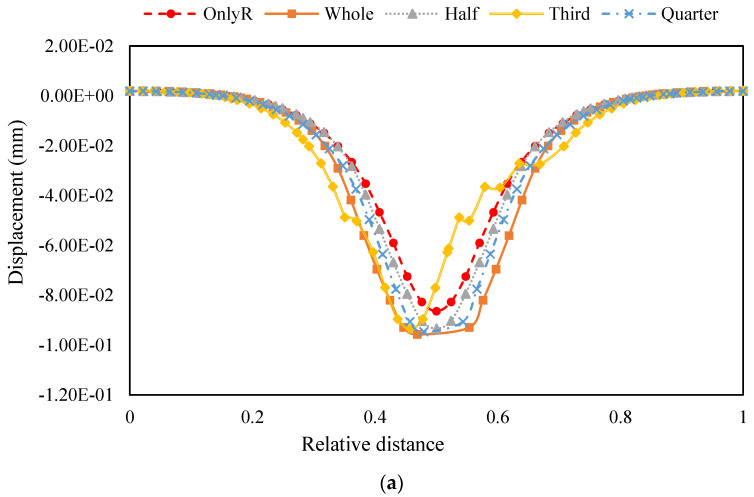

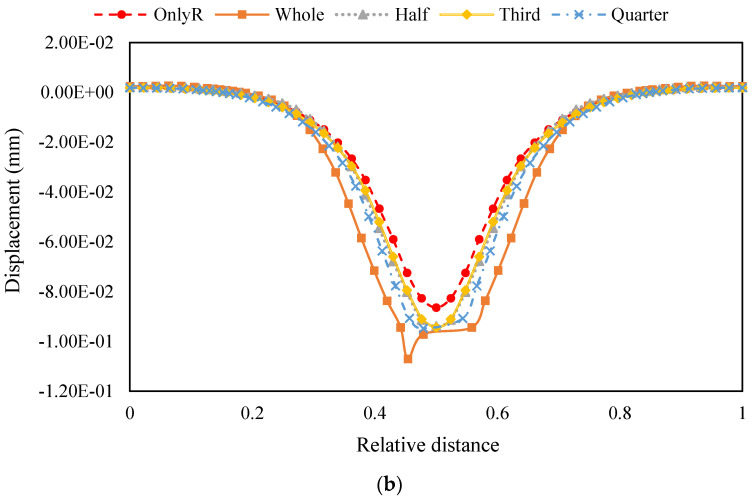
Displacement results of asphalt mixture layer bottom. (**a**) One tyre placed; (**b**) three tyres placed.

Accordingly, the structural displacement analysis data were extracted for the centreline, which was located at the bottom of the asphalt mixture, perpendicular to the direction of travel. The displacement image is plotted in Figure 7.

A comparison of Figure 7a,b demonstrates that the number of tyres placed exerts negligible influence on the outcomes of the surface layer bottom displacements. This finding is corroborated by the comparison of Figure 6, which demonstrates that the effect of the number of tyres is predominantly discernible in the cement-stabilised gravel layer. However, both Figure 7a,b demonstrate a phenomenon in which the pavement structure with tyres exhibits a more homogeneous settlement change at the location of the loads. For the original pavement structure, the displacement of bottom mixture layer approximates a standard parabola. However, for the pavement structure with tyres placed, a sudden change in displacement at the maximum value of the displacement is not evident. Instead, a smoother transition state was observed, which was most apparent when a whole tyre was placed, and the states of the structures placed one-half and one-quarter tyres were similar. This tyre placement has the potential to mitigate the unevenness of the road surface, resulting from rutting in the mixture, thereby enhancing driving comfort. However, the effect of cushioning displacement is not evident when one-third of the tyres are placed.

#### 5.1.2. Structural Stress Analysis for Static Loads

**Figure 8:**
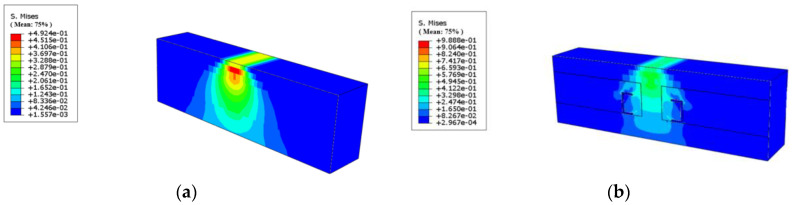

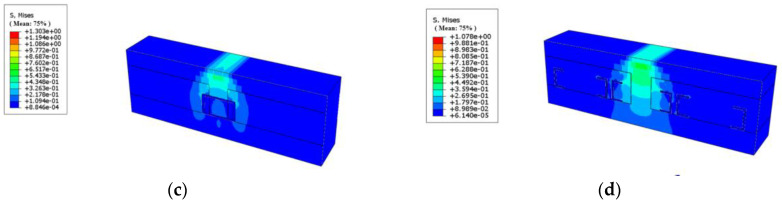
Stress cloud of some pavement structures. (**a**) Only road (OnlyR); (**b**) one whole tyre placed; (**c**) one 1/4 tyre placed; (**d**) three whole tyres placed.

The stress cloud of the pavement structure is shown in Figure 8.

A comparison of Figure 8a with the other images reveals that the presence of tyres significantly impacts the structural stress distribution. In the depth direction, tyres cause stresses to spread outwards. In the plane of the direction of travel, tyres constrict stresses, with the proximity of the tyre carcass affecting this constricting effect. This results in a tendency for the stress distribution lines near the tyre to be closer to the tyre on the stress cloud diagram, which, to a certain extent, demonstrates the stress absorption function of the tyre. As illustrated in Figure 8b,c, the stress undergoes a transition from the placement centre to the direction close to the tyre carcass following the placement of the tyre. Under identical loading conditions, the stress at the tyre’s centre is invariably less than the road stress of the bulk on both sides. This observation signifies that the rubber tyre exerts the hoop effect, and the closer to the centre of the composite, the less affected it is by the hoop effect of the rubber tyre, and the larger the value of the road stress. A comparison of Figure 8b,d reveals that the number of tyres has a negligible effect on the maximum and minimum values of stresses under these loading conditions. However, it is evident that placing more than one tyre at the same time results in a more pronounced stress absorption phenomenon in the adjacent tyre proximity area, manifesting as a more evident effect of tyre radial constriction.

**Figure 9:**
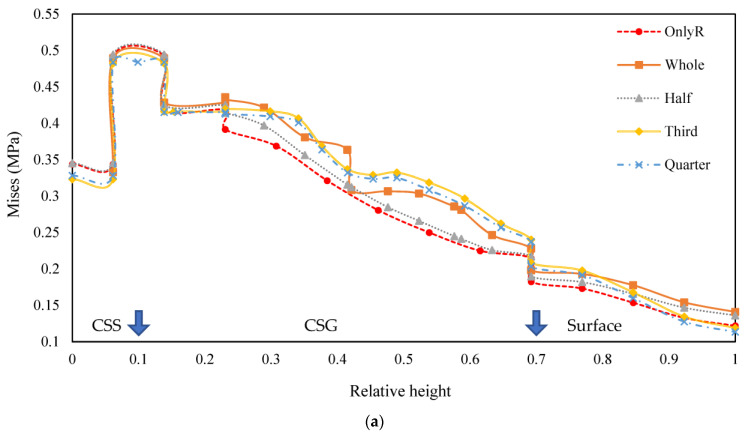

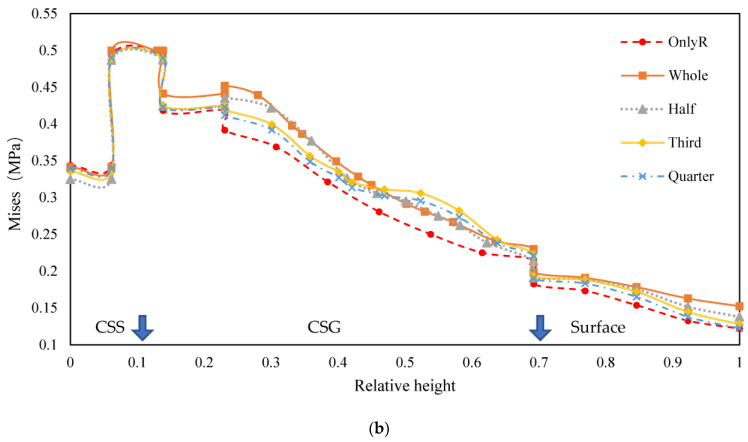
Equivalent force variation curves in the depth direction. (**a**) One tyre placed; (**b**) three tyres placed. Note: arrow positions represent the location of structural layer changes.

The variation of equivalent force along the depth direction for different structures was drawn, as illustrated in Figure 9.

As depicted in Figure 9a,b, the stress distribution maps reveal that the inclusion of tyres significantly alters the stress propagation within the pavement structure. Specifically, the presence of tyres induces an outward dispersion of stresses along the depth axis, as indicated by the elevated structural equivalent stresses in the cement-stabilised gravel layer compared to the original pavement. This phenomenon underscores the role of tyres in promoting a more uniform stress distribution across the pavement layers, effectively reducing abrupt stress variations that typically arise from material transitions. Moreover, the analysis demonstrates that the uniformity of stress distribution is largely independent of tyre size, suggesting that tyres of varying dimensions can similarly enhance the structural integrity of the pavement. Notably, the introduction of three tyres results in a more gradual stress curve, which may be attributed to the improved homogeneity of the model’s mesh configuration. The radial stress changes induced by the tyres are predominantly localised within the cement-stabilised gravel layer, with the most pronounced effects observed in configurations incorporating three tyres.

**Figure 10:**
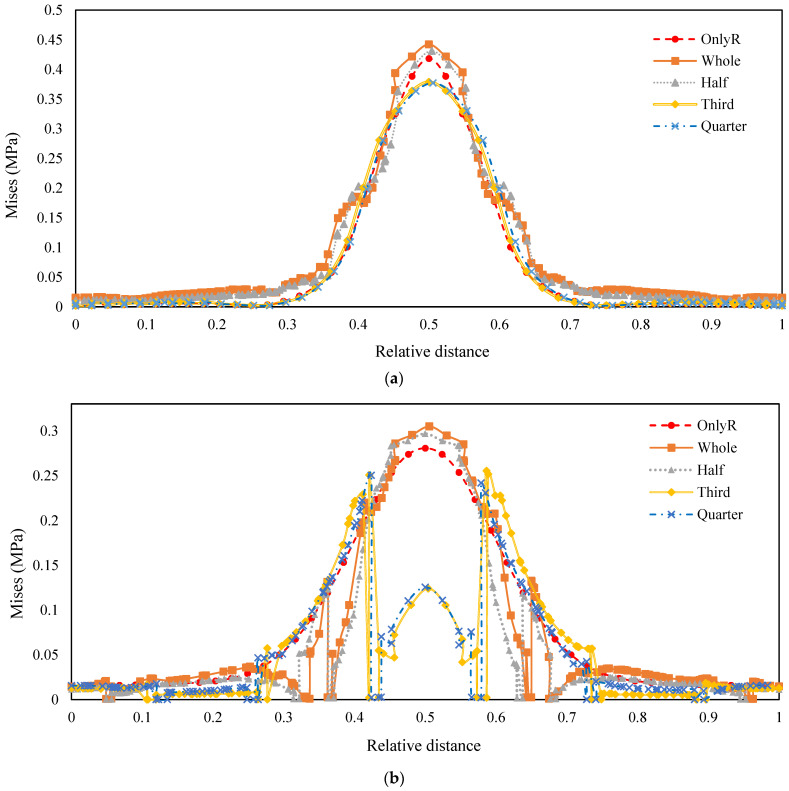

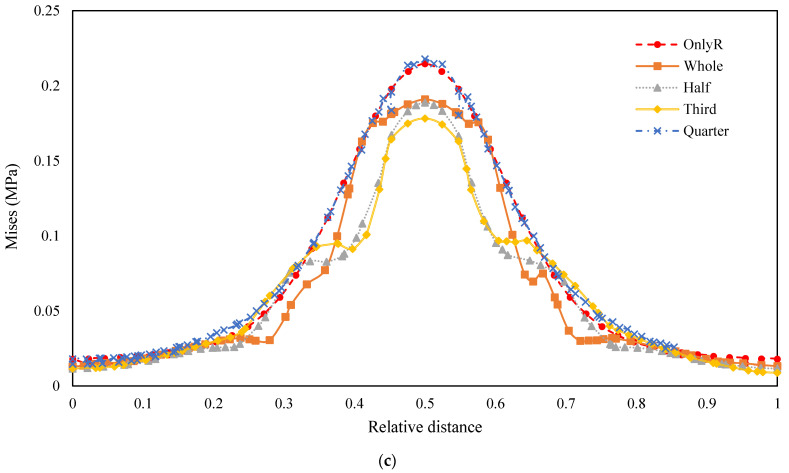
Equivalent stress distribution results for Static Loads. (**a**) Top; (**b**) middle; (**c**) bottom.

To further elucidate these findings, the equivalent stress variations at the top surface, middle plane, and bottom surface of the cement-stabilised gravel layer were meticulously analysed, as illustrated in Figure 10.

As shown in Figure 10a, the equivalent stress variations in the medial segment further confirm that the presence of tyres facilitates stress propagation along the depth direction. At relative distances of approximately 0.4 and 0.6, the equivalent stress values in this region are notably lower than those in the original pavement structure. This reduction is attributed to the hoop effect generated by the tyre carcass projection, which effectively mitigates stress concentrations in the pavement. For structures incorporating one-third and one-quarter tyres, the maximum equivalent stress at the stress diffusion location remains lower than that of the original pavement. This is because the selected centreline position is closer to the tyre carcass projection, where the hoop effect is most pronounced.

Figure 10b illustrates that the inclusion of one-third and one-quarter tyres within the middle interface of the cement-stabilised gravel layer leads to a significant reduction in stress. This is due to the proximity of the tyre carcasses to the middle cement interface, which amplifies the hoop effect and consequently reduces equivalent stresses within the road structure. However, abrupt stress changes are observed on both sides of the stress reduction curve, which result from the intersection points with the tyre boundary during the modelling process. These stress concentrations are artifacts of the simplified curve modelling and are unrelated to the intrinsic properties of the tyres. Nevertheless, these effects can be accurately represented through refined modelling techniques. Additionally, for structures with whole and half tyres, the equivalent stresses in the central region (0.45–0.55) are slightly higher than those in the original structure, while stresses near the inner tyre region are lower. This indicates that the closer the proximity to the tyre carcass, the more pronounced the hoop effect, thereby enhancing the road’s load-bearing capacity. 

In Figure 10c, the equivalent stress of the structure exhibits significant fluctuations at relative distances of 0.4 and 0.6 from the road surface. These fluctuations are likely due to the distribution range of contact points between the tyre base and the road structure, as well as the interaction and deformation conditions of the tyre. Except for the one-quarter tyre curve, which closely aligns with the original road surface, the trends in the curves demonstrate that tyres contribute to a more uniform stress distribution within the road structure. For road engineering applications, the uniformity of stress distribution is of greater significance than the absolute maximum or minimum stress values. This finding further underscores the beneficial role of tyre placement in optimising road load distribution and enhancing structural performance.

Across the three positions, the top surface is most directly influenced by the hoop effect, leading to significant stress reduction near the tyre carcass projection. The middle plane, while also benefiting from the hoop effect, exhibits a more complex stress distribution due to its intermediate position and interaction with tyre boundaries. The bottom surface, on the other hand, is primarily affected by the load transfer mechanisms and tyre deformation, resulting in stress fluctuations rather than uniform reduction. Overall, the incorporation of tyres enhances stress uniformity across all layers, with the most pronounced effects observed at the top surface and middle plane. This layered analysis underscores the importance of considering the tyre placement and size in pavement design to optimise stress distribution and improve structural performance.

### 5.2. Simulated Results Under Moving Constant Loads

#### 5.2.1. Selection of Data Observation Points

**Figure 11:**
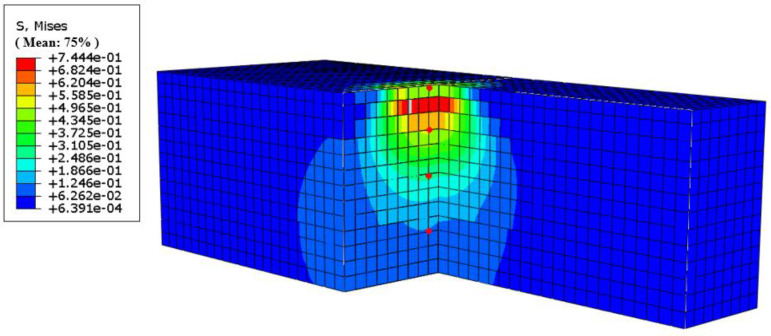
Schematic of data observation positions.

In contradistinction to static analysis, moving loads are time dependent, and the mechanical response of the structure must be analysed at a particular point. For the original road structure, the centre point of the selected location of the mechanical index is selected as the data point, and for the structure containing tyres, the data point is taken on the vertical axis corresponding to the centre of the tyre. The calculation of the road structure necessitates the selection of five points for each structure, as illustrated by the red dots highlighted in the schematic in Figure 11. Due to variations in the location of the corresponding centre points for different tyre sizes, the one-third and one-quarter tyre structures take points closer to the carcass, resulting in different peak indicator locations for different structures.

#### 5.2.2. Structural Displacement Analysis for Moving Loads

**Figure 12:**
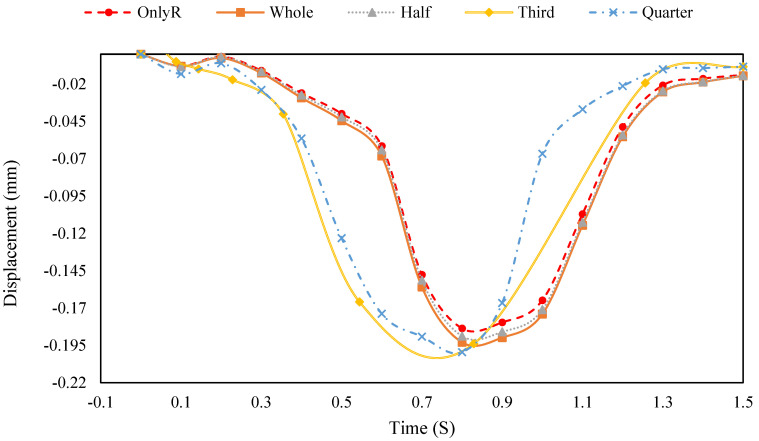
Displacement results of the top surface of the road.

The top surface of the pavement was selected for displacement analysis, and the outcomes are demonstrated in Figure 12.

As illustrated in Figure 12, the maximum displacement of the pavement structure incorporating tyres is greater than that of the original pavement structure. This observation can be explained by the fact that the selected numerical measurement point is located at the centre of the tyre placement, which coincides with the concentration area of equivalent force distribution. As a result, the displacement at this point is amplified compared to the original pavement structure. However, the data recorded after 1.5 s—when the loads have completely left the surface of the structure—reveal that the residual deformation of the tyre-reinforced pavement is less than that of the original pavement. This behaviour is likely due to the superior elastic properties of tyre rubber compared to traditional aggregate materials. The findings suggest that while the tyre-reinforced structure exhibits greater initial sensitivity to load application, the hyperelastic nature of the rubber material enables it to recover more effectively from deformation. This enhanced resilience ultimately leads to a reduction in permanent deformation, highlighting the potential benefits of incorporating tyres into pavement structures for improved durability and performance under dynamic loading conditions.

#### 5.2.3. Structural Stress Analysis for Moving Loads

**Figure 13:**
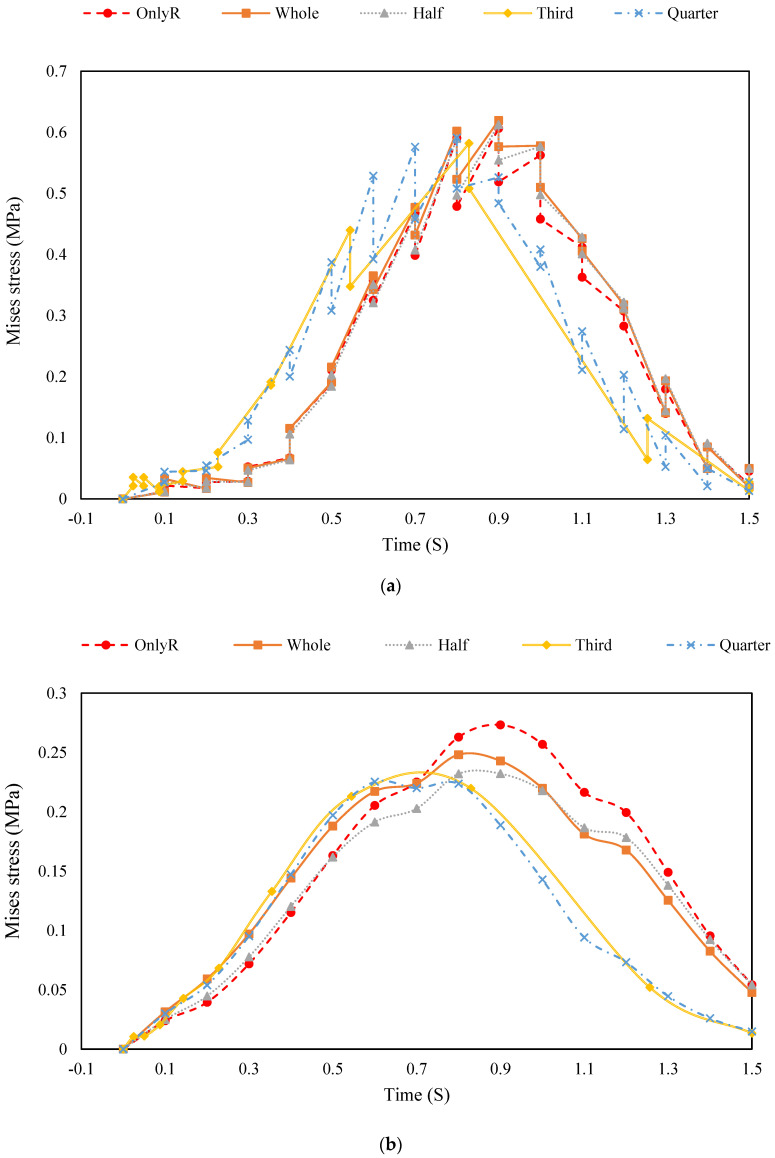

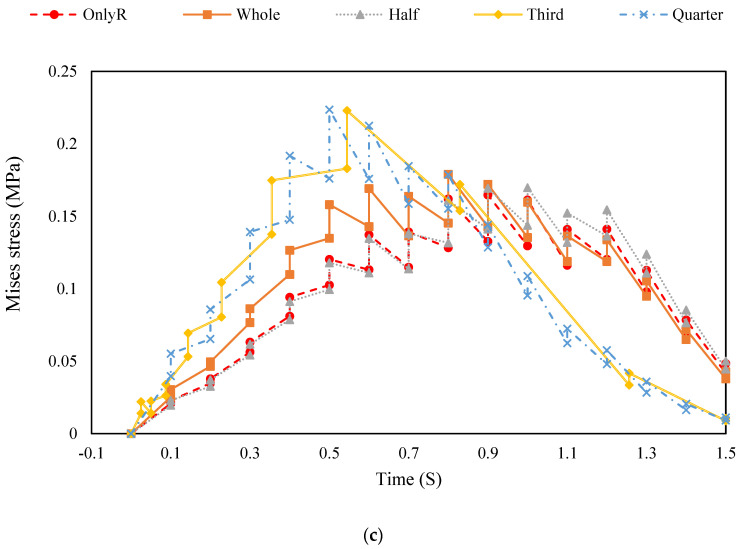
Equivalent stress distribution results for Moving Loads. (**a**) Top; (**b**) middle; (**c**) bottom.

Equivalent stress results for the top, midplane, and bottom surfaces of the cement-stabilised gravel layer are plotted in Figure 13.

As shown in Figure 13a, the influence of the tyre structure on the top surface of the cement-stabilised gravel layer is minimal, as indicated by the equivalent force distribution. However, the variability in the timing of peak stress appearance can be attributed to the differing positions of the centre points for tyres of varying sizes. This results in distinct peak positions for different tyre configurations. For instance, smaller tyres (one-third and one-quarter) exhibit peak stresses at different times compared to larger tyres (one-half or whole tyres), reflecting the localised effects of tyre placement on stress distribution.

In Figure 13b, the hoop effect of the tyres on the midplane is evident. For pavement structures incorporating one-third and one-quarter tyres, the measurement points are closer to the tyre carcass, making the hoop effect more pronounced. This is reflected in the smaller peak equivalent stresses observed in these configurations. In contrast, for structures with one-half tyres, the measurement points are farther from the carcass, reducing the perceptibility of the hoop effect. Nevertheless, the equivalent stresses in all tyre-reinforced structures remain lower than those in the original pavement, underscoring the stress-mitigating benefits of tyre incorporation.

Figure 13c reveals that the original pavement structure exhibits the smallest peak equivalent stress at the base. For pavement structures containing whole or one-half tyres, the equivalent stress curves largely overlap with those of the original pavement, indicating a similar stress distribution pattern. However, for structures with one-third or one-quarter tyres, the overlap is less pronounced, and the equivalent stress changes more rapidly. This suggests that the stress-spreading effect of the rubber material diminishes sooner in smaller tyre configurations, leading to a quicker return to baseline stress levels.

The results highlight the varying influence of tyre size and placement on the stress distribution across different layers of the pavement structure. While the top surface is minimally affected, the midplane demonstrates a clear hoop effect, particularly for smaller tyres. At the base, larger tyres (whole and one-half) exhibit stress distributions similar to the original pavement, whereas smaller tyres (one-third and one-quarter) show more rapid stress changes and reduced overlap. These findings emphasise the importance of tyre size and placement in optimising stress distribution and enhancing the resilience of pavement structures. Smaller tyres may offer localised stress reduction benefits, while larger tyres provide more uniform stress distribution across the pavement layers.

### 5.3. Sensitivity Analyses of Pavement Structural Parameters

#### 5.3.1. Influence on the Maximum Deformation at the Bottom of the Surface Layer

**Figure 14:**
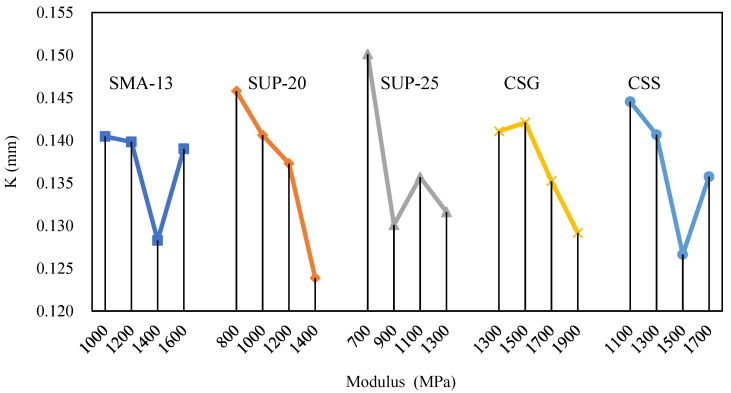
Impact of factors on MDBSL.

Based on the maximum values of the bottom deformation of the asphalt mixture obtained from each test program, the mean and extreme deviation for different levels of each factor were calculated. To visualise the relationship between each factor and the deformation index, a graph was plotted, as shown in Figure 14. In this graph, the factor levels are represented on the horizontal axis, while the mean values of the maximum bottom deformation, denoted as K, are plotted on the vertical axis.

As illustrated in Figure 14, there were significant differences in the effect of the modulus level of each structural layer on the test results. Among them, SUP-20 and SUP-25 have larger extreme differences, indicating that changes in the moduli of the middle and bottom layers within the test range lead to more significant numerical changes in the bottom deformation of the surface layer. Therefore, the moduli of SUP-20 and SUP-25 are the most significant influence factors on the test results. The sensitivity of MDBSL to the modulus factor of each structural layer was ranked as SUP-20 > SUP-25 > CSS > CSG > SMA-13, which shows that when the value of the deformation at the bottom of the surface layer is taken as an indicator of key concern, priority should be given to controlling the modulus parameter of the middle and lower layers of the pavement.

From the results of the mean value analysis, for the middle surface layer and the CSG layer, a larger modulus helps to reduce bottom deformation; however, for the top layer and the CSS layer, an excessively large modulus may lead to an increase in deformation. In addition, for the lower surface layer, a material with a modulus greater than 900 MPa should be selected in the structural design to ensure the stability and durability of the pavement structure.

#### 5.3.2. Influence on the Extreme Difference of Equivalent Stress in the Depth Direction

**Figure 15:**
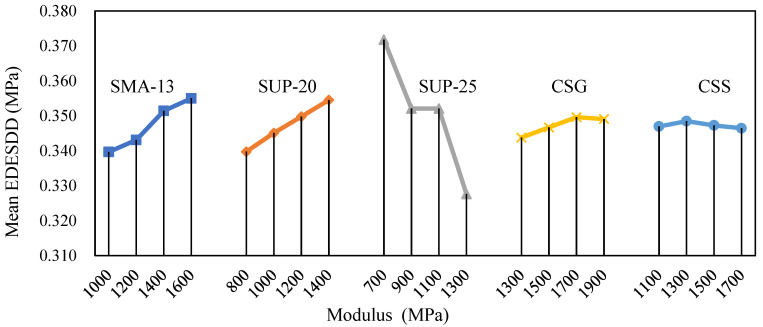
Impact of factors on EDESDD.

To analyse the influence of each factor on the equivalent force in the depth direction, EDESDD values under each test protocol were calculated. The relationship between each factor and the mean EDESDD is illustrated in Figure 15. In this figure, the factor levels are plotted on the horizontal axis, while the arithmetic mean values of EDESDD are represented on the vertical axis. The magnitude of EDESDD serves as an indicator of the uniformity of stress distribution within the pavement structure. A smaller extreme difference suggests better continuity in force transmission, a more balanced structural response, and a more uniform settlement.

From Figure 15, it can be observed that the extreme differences for the SUP-25 and SMA-13 factors are relatively large, with SUP-25 exhibiting the most significant variation. This indicates that changes in the moduli of the lower layer (SUP-25) and the top layer (SMA-13) within the tested range lead to substantial changes in the extreme difference of equivalent stress values. Therefore, the moduli of SUP-25 and SMA-13 are the most influential factors on the EDESDD FEM test results, making them the most critical parameters. The sensitivity of the extreme difference in equivalent stress to each factor, ranked from highest to lowest, is as follows: SUP-25 > SMA-13 > SUP-20 > CSG layer > CSS. Consequently, when aiming for a more uniform settlement, priority should be given to controlling the modulus parameters of the middle and upper layers. In terms of the magnitude of the mean values, SUP-25 exhibits the largest variation, further underscoring the importance of the lower layer in force transmission within the structure.

Overall, while a high-modulus structure can reduce structural deformation to some extent, it may also lead to uneven stress distribution. The modulus parameter of the underlying layer (SUP-25) plays a particularly significant role in pavement structures subjected to tyre loading, both in terms of the maximum deformation at the bottom of the asphalt mixture and the extreme difference in equivalent stresses in the depth direction. This highlights the need for careful consideration of the lower layer’s modulus in pavement design to ensure balanced stress distribution and uniform settlement.

### 5.4. Integrated Analysis

#### 5.4.1. Stress Distribution and Deformation

The stress distribution and deformation results collectively demonstrate that tyre reinforcement enhances the structural performance of pavements in several ways, as follows:

(1) Stress redistribution and tyre reinforcement:

The stress distribution data (Figure 13a–c) demonstrate that tyre reinforcement, particularly with smaller tyres (one-third and one-quarter), enhances stress uniformity by redistributing stresses outward through the hoop effect. This aligns with the sensitivity analysis, which highlights the importance of the middle and lower layers (SUP-20 and SUP-25) in stress mitigation. The reduced peak equivalent stresses in tyre-reinforced structures further support the idea that tyre incorporation can complement the role of high-modulus layers in achieving uniform stress distribution.

(2) Deformation control and resilience:

The deformation data (Figure 12) show that tyre-reinforced structures exhibit greater initial displacement but significantly less residual deformation compared to the original pavement. This resilience is attributed to the hyperelastic properties of tyre rubber, which enhance the structure’s ability to recover from deformation. The sensitivity analysis reinforces this finding by emphasising the importance of the lower layer (SUP-25) in controlling maximum deformation. A high-modulus lower layer combined with tyre reinforcement can further enhance deformation resistance and structural durability.

(3) Balancing modulus and tyre reinforcement:

While a high-modulus structure can reduce deformation, it may also lead to stress concentration and uneven distribution. Tyre reinforcement addresses this issue by promoting stress uniformity and providing additional resilience. The combined use of high-modulus layers (particularly SUP-25) and tyre reinforcement offers a balanced solution for optimising both deformation control and stress distribution. This approach is especially beneficial for pavements subjected to dynamic or repeated loading.

(4) Priority areas for modulus control:

The middle (SUP-20) and lower (SUP-25) layers should be prioritised in pavement design, as they have the greatest influence on deformation and stress distribution. For the top layer (SMA-13), a moderate modulus is recommended to avoid excessive deformation while maintaining stress uniformity.

(5) Tyre reinforcement strategies:

Smaller tyres (one-third and one-quarter) are more effective in localised stress reduction and enhancing the hoop effect, making them suitable for areas with high-stress concentrations. Larger tyres (one-half or whole tyres) provide a more uniform stress distribution and are better suited for broader areas requiring balanced load transfer.

(6) Holistic design approach:

A holistic design approach that integrates high-modulus layers with tyre reinforcement can optimise pavement performance by balancing deformation control, stress uniformity, and resilience. Future research should explore the long-term performance of such designs under varying traffic and environmental conditions to validate their effectiveness.

#### 5.4.2. Model Limitations and Their Potential Impact on Real-World Applicability

The assumptions of no sliding or separation between pavement layers and the exclusion of construction stress and temperature effects significantly simplify the numerical model. While these assumptions facilitate the computational analysis and provide valuable insights into the stress distribution and deformation behaviour of tyre-reinforced pavements, they may limit the model’s applicability to real-world conditions.

(1) No sliding or separation between layers

The model assumes perfect bonding between pavement layers, meaning there is no relative movement (sliding) or detachment (separation) at the interfaces. In reality, the interfacial behaviour between layers can be complex, influenced by factors such as material compatibility, surface roughness, and loading conditions. In real pavements, poor bonding or interfacial sliding can lead to stress concentration at the layer boundaries, potentially causing premature cracking or delamination. The model’s assumption of perfect bonding may underestimate these localised stresses, leading to overly optimistic predictions of structural performance. Sliding or separation between layers can alter the deformation patterns, particularly under dynamic or heavy loading. The model’s inability to capture these effects may result in inaccurate predictions of pavement deflection and resilience. Over time, environmental factors (e.g., moisture infiltration and freeze–thaw cycles) can exacerbate interfacial issues, further compromising pavement durability. The model’s simplified assumptions may not account for these long-term degradation mechanisms. Future studies could incorporate interfacial friction models or cohesive zone elements to simulate sliding and separation behaviours. Experimental validation of interfacial properties under varying conditions would also enhance the model’s realism.

(2) Exclusion of construction stress

The model does not account for stresses induced during the construction process, such as compaction forces, uneven layer thickness, or initial settlement. These stresses can influence the initial state of the pavement and its subsequent performance under traffic loading. Construction-related stresses can cause initial deformation or uneven settlement, which may not be captured by the model. This could lead to discrepancies between predicted and actual deformation behaviours. The exclusion of construction stresses may result in an incomplete understanding of stress redistribution within the pavement structure, particularly in the early stages of its service life. Variability in construction quality (e.g., compaction density and layer uniformity) can significantly affect pavement performance. The model’s idealised conditions may not reflect the variability observed in real-world applications. Incorporating construction-induced stresses into the model, possibly through initial stress fields or probabilistic analysis, could improve its accuracy. Field measurements during construction could also provide valuable data for model calibration.

(3) Exclusion of temperature effects

The model ignores the influence of temperature variations on the material properties and structural behaviour. Temperature changes can affect the modulus of elasticity, thermal expansion, and stress–strain relationships of pavement materials, particularly tyre-derived rubber. Temperature fluctuations can cause significant changes in the mechanical properties of rubber and other pavement materials. For example, rubber becomes stiffer at low temperatures and softer at high temperatures, which may alter stress distribution and deformation patterns. Temperature gradients within the pavement can induce thermal stresses, leading to cracking or warping. The model’s exclusion of these effects may underestimate the risk of thermal-related damage. Pavement performance can vary seasonally due to temperature changes. The model’s inability to account for these variations may limit its applicability in regions with extreme climates. Future work could incorporate temperature-dependent material properties and thermal stress analysis into the model. Experimental studies on the thermal behaviour of tyre-reinforced pavements would provide valuable insights for model refinement.

#### 5.4.3. Effects of Tyre Compositions on Performance

The composition of tyres, including the type of rubber, additives, and ageing effects, plays a critical role in determining the performance of tyre-reinforced structures. Understanding these factors is essential for optimising material selection, design, and long-term durability. Future research should focus on experimental studies to characterise the mechanical and ageing behaviours of different tyre compositions, as well as the development of strategies to mitigate ageing effects and enhance performance. By addressing these aspects, tyre-reinforced pavements can be designed to achieve superior performance and sustainability in real-world applications.

(1) Rubber types and their mechanical properties

Tyres are typically composed of various types of rubber, including natural rubber (NR), styrene–butadiene rubber (SBR), and polybutadiene rubber (BR), each with distinct mechanical properties. The type of rubber used in tyres can influence the stress distribution within the pavement structure. For example, rubbers with higher elasticity (e.g., NR) may provide better stress redistribution, while those with higher stiffness (e.g., SBR) may offer greater load-bearing capacity. The viscoelastic properties of rubber affect the deformation and recovery behaviours of tyre-reinforced structures. Rubbers with higher resilience (e.g., NR) may reduce permanent deformation, while those with higher stiffness (e.g., SBR) may limit initial deflection.

(2) Additives and their role in performance

Tyre rubber often contains additives such as carbon black, silica, and vulcanising agents, which modify its properties. Carbon black can enhance the strength, abrasion resistance, and UV stability of rubber. However, it may increase its stiffness, potentially reducing the material’s ability to absorb energy. Silica can improve wet traction and reduce rolling resistance, but it may also affect the rubber’s thermal and mechanical properties. Vulcanising agents can improve the cross-linking of rubber molecules, enhancing durability and resistance to deformation. Additives like carbon black and silica can improve the long-term durability of tyre-derived materials by enhancing the resistance to wear, ageing, and environmental degradation. The thermal conductivity and heat resistance of rubber can be influenced by additives, affecting the material’s performance under temperature variations.

(3) Ageing effects on tyre materials

Ageing is a critical factor that can alter the properties of tyre rubber over time, affecting the performance of tyre-reinforced structures. Aged rubber may exhibit reduced resilience, leading to increased permanent deformation and decreased load-bearing capacity. Ageing can cause microcracks and interfacial debonding, reducing the structural integrity of tyre-reinforced pavements. The long-term performance of tyre-reinforced structures may be compromised if ageing effects are not adequately addressed in the design and material selection process.

#### 5.4.4. Waste Utilisation and Environmental Analysis

The use of whole tyres in a cement-stabilised gravel base (300 mm thick), which is equal to a proportion of 1.2% by mass, offers significant advantages compared to traditional rubber powder-modified asphalt or asphalt mixtures.

In terms of the amount of waste utilised, using whole tyres in the base layer achieves a tyre mass proportion of 1.2%, meaning a larger amount of waste tyres can be consumed per ton of material. In contrast, rubber powder-modified asphalt typically has a lower rubber powder content (usually less than 1%), resulting in a lower waste utilisation rate.

During the production and construction phases, the use of whole tyres eliminates the need for additional processing steps (such as shredding tyres into rubber powder), significantly reducing energy consumption. It also avoids environmental pollution issues like dust and noise generated during tyre shredding. Moreover, the integrity of whole tyres minimises the release of harmful substances during use, while rubber powder-modified asphalt may release volatile organic compounds (VOCs) during construction and use, causing secondary environmental pollution. Since whole tyres are directly used in the base material, no high-temperature heating or complex processes are required during construction, reducing energy consumption and pollutant emissions. Furthermore, whole tyres do not release harmful gases at high temperatures, unlike rubber powder-modified asphalt, further minimising environmental pollution during use.

In terms of the full life cycle of road use, the use of whole tyres in the base layer preserves their integrity, preventing the generation of microplastics or fine particles during long-term use. This reduces the risk of soil and water contamination. In contrast, rubber powder-modified asphalt may degrade and wear, producing microplastics that can be washed into soil and water bodies by rainwater, causing long-term ecological harm.

Above all, the use of whole tyres in the base layer demonstrates significant advantages over traditional rubber powder-modified asphalt or TDA mixtures. Whole-tyre utilisation not only increases the consumption of waste tyres but also reduces energy consumption and environmental pollution, particularly by avoiding the release of harmful substances during construction and use. Therefore, whole-tyre utilisation is more sustainable and environmentally beneficial in waste management and environmental protection.

## 6. Conclusions, Limitations, and Further Research Recommendations

### 6.1. Conclusions

In this study, numerical simulations based on the Abaqus finite element model were conducted to investigate the effects of incorporating waste tyres into the base layer (WTiBL). The following key conclusions were drawn:

(1) The placement of waste tyres in the base layer increases the transient settlement of the pavement structure compared to the original design, making WTiBL less suitable for heavy-load road applications. However, the tyre-reinforced structure exhibits superior deformation resilience, with a stronger ability to recover from deformation due to the hyperelastic properties of tyre rubber. This enhanced resilience highlights the potential of tyre-reinforced pavements for applications where deformation recovery is critical.
(2) For uniform settlement, the entire tyre stacking scheme is recommended, as it provides the most consistent stress distribution and minimises differential settlement. If minimising displacement is the primary concern, the one-third tyre configuration is preferred, although further research is needed to optimise the spacing between tyres. From a resource utilisation perspective, the one-half tyre configuration offers a balanced solution for structures with moderate requirements for both uniform settlement and displacement control.
(3) The inclusion of tyres in the base layer increases the equivalent stress within the CSG structure beneath the tyre. However, this effect diminishes as the size of the placed tyres decreases. Additionally, the projected portion of the tyre tread enhances the bearing capacity of the CSG structure, demonstrating the potential of tyre reinforcement to improve load distribution and structural performance.
(4) The middle (SUP-20) and bottom (SUP-25) layers should be prioritised in the WTiBL design, as they have the greatest influence on deformation and stress distribution. A moderate modulus is recommended for the surface course (SMA-13) to avoid excessive deformation while maintaining stress uniformity. The combined use of high-modulus layers (particularly SUP-25) and tyre reinforcement provides a balanced solution to optimise both deformation control and stress distribution.
(5) The use of waste tyres in pavement structures provides a sustainable solution for tyre disposal. However, the long-term environmental and economic benefits, as well as the effects of tyre composition and ageing, require further investigation to optimise the design and implementation of tyre-reinforced pavements.

### 6.2. Limitations and Further Research Recommendations

Limitations: (1) The study assumes no sliding or separation between pavement layers and ignores construction stresses and temperature effects, which may oversimplify real-world conditions. (2) The model treats tyre materials as homogeneous, which may not fully capture the variability in rubber properties and their impact on structural behaviour. (3) The findings are based on numerical simulations, and the lack of experimental validation limits the generalisability of the results. (4) The study provides a qualitative discussion of environmental benefits but does not include a detailed economic feasibility analysis or life-cycle assessment. (5) While the theoretical and numerical results are promising, the scalability of the methodology to large-scale applications requires further investigation through pilot projects and case studies.

Further research recommendations: (1) Conduct laboratory and field experiments to validate the numerical findings, particularly the stress distribution and deformation behaviour of tyre-reinforced pavements under dynamic loading conditions. (2) Investigate the long-term performance of tyre-reinforced pavements under varying environmental conditions, such as temperature fluctuations, moisture exposure, and UV radiation, to assess durability and ageing effects. (3) Explore the use of different tyre sizes, shapes, and arrangements (e.g., layered vs. mixed configurations) to optimise stress distribution and structural performance. (4) Study the interfacial behaviour between tyre-derived materials and the surrounding pavement layers, including the effects of sliding, separation, and bonding strength, to refine the model assumptions. (5) Perform a comprehensive life-cycle assessment (LCA) to evaluate the environmental and economic impacts of tyre-reinforced pavements compared to conventional materials, considering factors such as material production, construction, maintenance, and end-of-life disposal. (6) Implement projects in real-world settings to assess the feasibility, performance, and cost effectiveness of tyre-reinforced pavements under actual traffic and environmental conditions.

## Figures and Tables

**Table 1:** Road structure parameters.

Structural Layer	Thickness/mm	Modulus of Elasticity (*E)*/MPa	Poisson’s Ratio (ν)
SMA-13	40	1200	0.28
SUP-20	50	1000	0.28
SUP-25	60	900	0.3
Cement-stabilised gravel (CSG)	300	1500	0.25
Cement-stabilised soil (CSS)	200	1300	0.26

**Table 2:** Road structure parameter design for factors analysis.

Level	Factors				
	Modulus of SMA-13 (MPa)	Modulus of SUP-20 (MPa)	Modulus of SUP-25 (MPa)	Modulus of Cement-Stabilised Gravel Layer (MPa)	Modulus of Cement-Stabilised Soil (MPa)
1	1000	800	700	1300	1100
2	1200	1000	900	1500	1300
3	1400	1200	1100	1700	1500
4	1600	1400	1300	1900	1700

**Table 3:** Orthogonal experimental design.

No.	SMA-13 (MPa)	SUP-20 (MPa)	SUP-25 (MPa)	Modulus of Cement-Stabilised Gravel Layer (MPa)	Modulus of Cement-Stabilised Soil (MPa)
1	1000	800	700	1300	1100
2	1000	1000	900	1500	1300
3	1000	1200	1100	1700	1500
4	1000	1400	1300	1900	1700
5	1200	800	900	1700	1700
6	1200	1000	700	1900	1500
7	1200	1200	1300	1300	1300
8	1200	1400	1100	1500	1100
9	1400	800	1100	1900	1300
10	1400	1000	1300	1700	1100
11	1400	1200	700	1500	1700
12	1400	1400	900	1300	1500
13	1600	800	1300	1500	1500
14	1600	1000	1100	1300	1700
15	1600	1200	900	1900	1100
16	1600	1400	700	1700	1300

## Data Availability

The original contributions presented in this study are included in the article. Further inquiries can be directed to the corresponding author.
